# HIV-1 genetic diversity and its distribution characteristics among newly diagnosed HIV-1 individuals in Hebei province, China

**DOI:** 10.1186/s12981-015-0087-2

**Published:** 2016-01-19

**Authors:** Xinli Lu, Cuiying Zhao, Wei Wang, Chenxi Nie, Yuqi Zhang, Hongru Zhao, Suliang Chen, Ze Cui

**Affiliations:** Hebei Provincial Center for Disease Control and Prevention, 97 Huaian East Rd, Yuhua District, 050021 Shjiazhuang, People’s Republic of China; College of Chemistry and Environmental Science, Hebei University, 180 Wusi East Rd, 071002 Baoding, People’s Republic of China

**Keywords:** HIV-1, Genetic diversity, Transmission, Distribution, Phylogeny, China

## Abstract

**Background:**

Since the first HIV-1 case in 1989, Hebei province has presented a clearly rising trend of HIV-1 prevalence, and HIV-1 genetic diversity has become the vital barrier to HIV prevention and control in this area. To obtain detailed information of HIV-1 spread in different populations and in different areas of Hebei, a cross-sectional HIV-1 molecular epidemiological investigation was performed across the province.

**Methods:**

Blood samples of 154 newly diagnosed HIV-1 individuals were collected from ten prefectures in Hebei using stratified sampling. Partial *gag* and *env* genes were amplified and sequenced. HIV-1 genotypes were identified by phylogenetic tree analyses.

**Results:**

Among the 139 subjects genotyped, six HIV-1 subtypes were identified successfully, including subtype B (41.0 %), CRF01_AE (40.3 %), CRF07_BC (11.5 %), CRF08_BC (4.3 %), unique recombinant forms (URFs) (1.4 %) and subtype C (1.4 %). Subtype B was identified as the most frequent subtype. Two URF recombination patterns were the same as CRF01_AE/B. HIV-1 genotype distribution showed a significant statistical difference in different demographic characteristics, such as source (*P* < 0.05), occupation (*P* < 0.05) and ethnicity (*P* < 0.05). The distributions of subtype B (*P* < 0.05), CRF01_AE (*P* < 0.05), CRF07_BC (*P* < 0.05) and subtype C (*P* < 0.05) showed significant differences in all ten prefectures, and the distributions of all six subtypes were significantly different in Shijiazhuang (*P* < 0.05) and Xingtai (*P* < 0.05), but not in other prefectures (*P* > 0.05). The differences in HIV-1 genotype distribution were closely associated with transmission routes. Particularly, all six subtype strains were found in heterosexuals, showing that HIV-1 has spread from the high-risk populations to the general populations in Hebei, China. In addition, CRF01_AE instead of subtype B has become the major strain of HIV-1 infection among homosexuals.

**Conclusions:**

Our study revealed HIV-1 evolution and genotype distribution by investigating newly diagnosed HIV-1 individuals in Hebei, China. This study provides important information to enhance the strategic plan for HIV prevention and control in China.

## Background

In 1985, four Chinese hemophiliac patients who accepted Factor VIII treatment were identified as having human immunodeficiency virus type one (HIV-1) [1]. In the following years, further HIV-1-positive individuals were found in provinces of mainland China [2–4]. Since 1985, the major drivers of HIV-1 prevalence in China have shifted from blood transmission to sexual contact transmission [5]. A nationwide molecular epidemiological investigation suggested this shift in HIV-1 transmission patterns in China and showed that HIV-1 had spread from the high-risk population through heterosexual transmission [6].

Hebei, with an area of 190,000 km^2^, is located in north China, surrounding Tianjin and Beijing, and neighboring Henan in the south. There are 11 prefectures in Hebei, including 172 counties and 2228 townships. Since the first HIV-1 case was reported in 1989, 3189 HIV/AIDS cases had been reported up to the end of 2012, accounting for 0.7 % of nationwide HIV/AIDS cases, which represented a low prevalence in Hebei compared with other areas of China. Initially, HIV-1 infections were mainly driven by blood transmission. Between 1993 and 1995, HIV-1 individuals were found among paid blood donors in many blood collection stations in Langfang, and many HIV-1 individuals were also found among blood recipients in Xingtai. In Hebei, an HIV-1 outbreak occurred through contaminated blood at that time [7, 8]. After 2005, the proportion of sexual transmission cases, rather than blood transmission cases, rose rapidly in Hebei [9], reaching 87.1 % in 2012. Moreover, HIV-1 subtypes prevalent in Hebei changed from CRF07_BC, subtype C and subtype B in 2002 [10] to CRF07_BC, subtype C, CRF01_AE, CRF08_BC and subtype B in 2008 [11]. In addition, there were different HIV-1 subtypes in different populations [12]. However, the HIV-1 genotype distribution characteristics among newly diagnosed individuals in Hebei are not known. Therefore, with the diversity of the transmission routes, it is necessary to perform a detailed and extensive analysis on HIV-1 genotypes and their distribution characteristics among newly diagnosed individuals. HIV-1 molecular epidemiological investigations are extremely important to estimate HIV-1 epidemic trends and to determine the real-time dynamics of HIV-1 genotypes to better prevent and control the rise of HIV-1 in this area. In this study, we focused on newly diagnosed HIV-1 individuals in Hebei to reveal HIV-1 genetic characteristics.

## Methods

### Participants

**Fig. 1:**
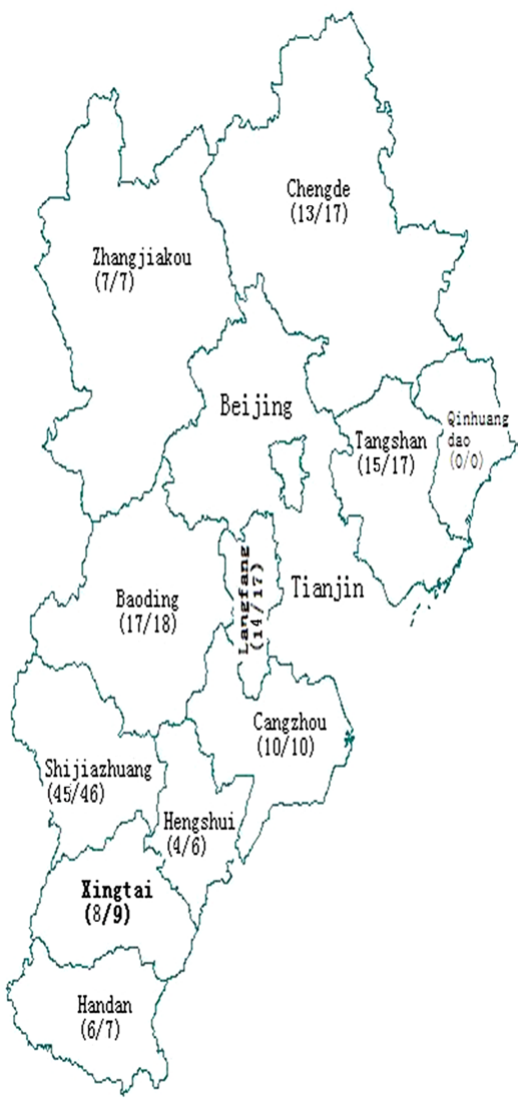
Geographical distribution of subjects collected from ten prefectures in this study. The numbers to the* left* and* right* of the “/” denote the subjects genotyped and the total subjects, respectively

The study was cross-sectional and used stratified sampling. Whole blood samples were collected from 154 newly diagnosed HIV-1 individuals who had not undergone treatment in ten prefectures of Hebei in 2011. Our study subjects were distributed in Shijiazhuang, Handan, Xingtai, Cangzhou, Chengde, Hengshui, Zhangjiakou, Tangshan, Baoding and Langfang (Fig. 1). Demographic data were collected via face-to-face interviews, using a standardized questionnaire when we collected their blood samples. Whole blood samples (50 μl) were used to detect CD4 cell counts with the FACSCount system (Becton and Dickinson Company, USA). Plasma samples separated from the whole blood were used to obtain HIV-1 RNA for subsequent analysis (HIV-1 RNA *gag* and *env* gene sequences).

### Ethics statement

In our study, informed medical consent was obtained from all adult patients and from parents/guardians of HIV-1-positive children before blood collection. Our study was approved by the local Ethics Committee at the Hebei Provincial Center for Disease Control and Prevention, according to the Helsinki II Declaration. The ethics board document number was IRB-2012004.

### Amplification of HIV-1 gene fragments

HIV-1 RNA was extracted from 140 μl of plasma using a High pure viral RNA kit (Qiagen, Valencia, CA, USA), followed by partial *gag* gene (HXB2: 781–1861) and *env* gene (HXB2: 7002–7541) amplification for HIV-1 genotyping. The *gag* gene fragment was amplified using One Step reverse transcription PCR (Takara, Dalian, China) with primers GAG-L (5′-TCGACGCAGGACTCGGCTTGC-3′) and GAG-E2 (5′-TCCAACAGCCCTTTTTCCTAGG-3′) in a 25 μl reaction volume. Cycling conditions were: one cycle of 50 °C for 30 min, 94 °C for 5 min, 55 °C for 1 min and 72 °C for 2 min; followed by 30 cycles of 94 °C for 30 s, 55 °C for 45 s and 72 ℃ for 1 min 30 s; and finally, 72 °C for 10 min, and holding at 4 °C. The nested *gag* PCR was implemented using 2 × Taq PCR MasterMix (Takara) and primers GUX (5′-AGGAGAGAGATGGGTGCGAGAGCGTC-3′) and GDX (5′-GGCTAGT TCCTCCTACTCCCTGACAT-3′) in a 50 μl reaction volume. Cycling conditions were: one cycle of 4 °C for 2 min, 55 °C for 1 min, 72 °C for 1 min 30 s; then 30 cycles of 94 °C for 30 s, 55 °C for 45 s and 72 °C for 1 min 30 s; and finally, 72 °C for 10 min, and holding at 4 °C. The *env* fragment was amplified with primers 44F(5′-ACAGTRCARTGYACACATGG-3′) and 35R (5′-CACTTCTCCAATTGT CCCTCA-3′) in a 25 μl reaction volume. Cycling conditions were: one cycle of 50 °C for 30 min, 94 °C for 5 min, 55 °C for 1 min and 72 °C for 2 min; then 30 cycles of 94 °C for 30 s, 55 °C for 45 s, and 72 °C for 1 min 30 s; and finally, 72 °C for 10 min, and holding at 4 °C holding. The nested *env* PCR was implemented using primers 33F (5′-CTGTTAAATGGCAGTCTAGC-3′) and 48R (5′-RATGGGAGGRGYATACA T-3′) in a 50 μl reaction volume. Cycling conditions were: one cycle of 94 °C for 2 min, 55 °C for 1 min, and 72 °C for 1 min 30 s; then 30 cycles of 94 °C for 30 s, 55 °C for 45 s, and 72 °C for 1 min 30 s; and finally, 72 °C for 10 min and holding at 4 °C for 10 min. Each step was performed with appropriate negative controls to detect possible contamination during the experiments. The PCR products were analyzed using 1 % agarose gel electrophoresis. Finally, positive amplicons isolated from agarose gels were sequenced by Biological Engineering Technology Services Ltd (Shanghai, China).

### Sequence analysis

All original sequence fragments in *gag* and *env* gene regions were edited and assembled into the whole sequences using Contig Express software 6.0 (InforMax, Inc.). All assembled sequences for the *gag* and *env* gene regions were aligned with the respective reference sequences from different areas using the Clustal W Multiple alignment and manual editing in BioEdit software 7.0. The phylogenetic trees were constructed using MEGA 5.0 with the neighbor-joining method and 1000 bootstrap replicates, based on the Kimura 2-parameter Model (MEGA version 5.0). According to the online jpHMM Program (http://jphmm.gobics.de/submission_hiv.html) and online RIP 3.0 (http://www.hiv.lanl.gov/content/sequence/RIP/RIP.html), the possible intertype mosaicisms of unassigned reading frames (URFs) were screened.

### Statistical analysis

Statistical analyses for this study were implemented using SPSS version 18.0 (SPSS Inc., Chicago, IL, USA). Means or frequencies of demographic data (age, CD4 cell counts) were calculated. Categorical variables were analyzed using a Chi squared test. When more than 20 % of cells had an expected count of less than five, Fisher’s exact test was used. All tests were two-sided, and a statistical result was considered significant when the *P* value was less than 0.05.

## Results

### Demographic characteristics of subjects

As indicated in Fig. 1, 154 newly diagnosed HIV-1 individuals were recruited from 10 of 11 prefectures in Hebei Province in 2011. Among them, 139 samples (90.3 %; 139/154) were successfully genotyped by combined phylogenetic tree analyses of *gag* and *env* gene sequences (Fig. 1). The 154 subjects recruited and 139 subjects genotyped showed no statistical differences in gender, age, CD4 counts, source, transmission routes, occupation, marital status, ethnicity and educational level (Table 1).

**Table 1:** The distribution of HIV-1 subjects successfully genotyped

Characteristic	Subjects	Subjects sequenced successfully	χ^2^	*P*
Total	154	139		
Gender			0.019	0.891
Male	113	101		
Female	41	38		
Age			0.239	0.971
≤18	8	8		
19–30	57	52		
31–40	48	40		
≥41	41	39		
CD4 count			0.053	0.974
≤200	54	47		
201–350	34	31		
≥351	66	61		
Source			0.530	1.000
Inpatient	27	25		
Preoperative detecting	17	13		
MSM	33	29		
VCT	14	13		
Blood	20	18		
Close contacts	23	23		
Pregnant women	4	4		
Female marriage immigrant	8	8		
Detained persons	8	6		
Transmission routes			0.272	0.992
Heterosexual	65	60		
Homosexual	57	52		
MTCT	7	6		
IDU	8	8		
Blood transfusion	17	13		
Occupation			0.257	0.968
Farmer	71	63		
Worker/clerk	49	47		
Student	15	14		
Commercial services	19	15		
Marital status			0.441	0.802
Married	91	81		
Unmarried	48	47		
Divorced/widowed	15	11		
Ethnicity			0.002	0.965
Han	139	125		
Minority	15	14		
Educational level			0.577	0.750
College and above	32	29		
Middle school	89	85		
Primary school and below	33	25		

*MSM* men who have sex with men, *VCT* Voluntary Counseling And Testing, *MTCT* mother-to-child, *IDU* intravenous drug injection

As shown in Table 2, among 139 subjects genotyped, the sex ratio of males to females was 1:0.38. The mean values of age and CD4 counts were 33.7 (2–70) years and 330.4 (1–1495) cells/μl, respectively. Sexual contact was the predominant transmission route and accounted for 80.6 % (112/139) of cases, including heterosexual contact (43.2 %, 60/139) and homosexual contact (37.4 %, 52/139), followed by blood transfusion (9.4 %), intravenous drug injection (IDU, 5.8 %) and mother-to-child (MTCT, 4.3 %). Their occupations included farmer (45.3 %, 63/139), worker/clerk (33.8, 47/139), commercial service (10.8 %, 15/139) and student (10.1 %, 14/139). Among the nine sources of transmission, men who have sex with men (MSM) constituted the main source (20.9 %, 29/139), followed by inpatient (18.0 %, 25/139), close contact (16.5 %, 23/139), blood donor (12.9 %, 18/139), and voluntary counseling and testing (VCT, 9.4 %, 13/139). Of 139 subjects, 58.3 % were married, 33.8 % were unmarried and 7.9 % were divorced/widowed. According to ethnicity, 89.9 % (125/139) of subjects were of Han ethnicity and the remaining 14 subjects were from minority nationalities, including Yi (5.0 %, 7/139), Man (2.2, 3/139), Hui (0.7 %, 1/139), Oroqen (0.7 %, 1/139), Uyghur (0.7 %, 1/139) and Dai (0.7 %, 1/139).

**Table 2:** Demographic distribution characteristics and HIV-1 genotypes

Characteristic	Subjects (%)	B (%)	CRF01_AE (%)	CRF07_BC (%)	CRF08_BC (%)	URFs (%)	C (%)	χ^2^	P
Total	139 (100.0)	57 (41.0)	56 (40.3)	16 (11.5)	6 (4.3)	2 (1.4)	2 (1.4)		
Gender								15.666	0.003
Male	101 (72.7)	38 (27.3)	49 (35.3)	10 (7.2)	3 (2.2)	1 (0.7)	0 (0.0)		
Female	38 (27.3)	19 (13.7)	7 (5.0)	6 (4.3)	3 (2.2)	1 (0.7)	2 (1.4)		
Age								29.655	0.002
≤18	8 (5.8)	5 (3.6)	1 (0.7)	1 (0.7)	0 (0.0)	0 (0.0)	1 (0.7)		
19–30	52 (37.4)	12 (8.6)	31 (22.3)	7 (5.0)	1 (0.7)	0 (0.0)	1 (0.7)		
31–40	40 (28.8)	18 (12.9)	12 (8.6)	6 (4.3)	4 (2.9)	0 (0.0)	0 (0.0)		
≥41	39 (28.1)	22 (15.8)	12 (8.6)	2 (1.4)	1 (0.7)	2 (1.4)	0 (0.0)		
CD4 count								225.734	0.001
≤200	47 (33.8)	31 (22.3)	9 (6.5)	3 (2.2)	3 (2.2)	1 (0.7)	0 (0.0)		
201–350	31 (22.3)	7 (5.0)	18 (12.9)	4 (2.9)	1 (0.7)	1 (0.7)	0 (0.0)		
≥351	61 (43.9)	19 (13.7)	29 (20.9)	9 (6.5)	2 (1.4)	0 (0.0)	2 (1.4)		
Source								69.241	<0.001^a^
Inpatient	25 (18.0)	15 (10.8)	6 (4.3)	2 (1.4)	1 (0.7)	1 (0.7)	0 (0.0)		
Preoperative detecting	13 (9.4)	5 (3.6)	5 (3.6)	1 (0.7)	2 (1.4)	0 (0.0)	0 (0.0)		
MSM	29 (20.9)	7 (5.0)	21 (15.1)	1 (0.7)	0 (0.0)	0 (0.0)	0 (0.0)		
VCT	13 (9.4)	7 (5.0)	6 (4.3)	0 (0.0)	0 (0.0)	0 (0.0)	0 (0.0)		
Blood donor	18 (12.9)	9 (6.5)	6 (4.3)	2 (1.4)	0 (0.0)	1 (0.7)	0 (0.0)		
Close contacts	23 (16.5)	13 (9.4)	6 (4.3)	2 (1.4)	1 (0.7)	0 (0.0)	1 (0.7)		
Pregnant women	4 (2.9)	0 (0.0)	3 (2.2)	0 (0.0)	1 (0.7)	0 (0.0)	0 (0.0)		
Female marriage immigrant	8 (5.8)	0 (0.0)	0 (0.0)	6 (4.3)	1 (0.7)	0 (0.0)	1 (0.7)		
Detained persons	6 (4.3)	1 (0.7)	3 (2.2)	2 (1.4)	0 (0.0)	0 (0.0)	0 (0.0)		
Transmission routes								49.215	<0.001^b^
Sexual contact								26.775	<0.001^c^
Heterosexual	60 (43.2)	28 (20.1)	16 (11.5)	9 (6.5)	4 (2.9)	2 (1.4)	1 (0.7)		
Homosexual	52 (37.4)	13 (9.4)	38 (27.3)	1 (0.7)	0 (0.0)	0 (0.0)	0 (0.0)		
MTCT	6 (4.3)	3 (2.2)	1 (0.7)	1 (0.7)	0 (0.0)	0 (0.0)	1 (0.7)		
IDU	8 (5.8)	0 (0.0)	1 (0.7)	5 (3.6)	2 (1.4)	0 (0.0)	0 (0.0)		
Blood transfusion	13 (9.4)	13 (9.4)	0 (0.0)	0 (0.0)	0 (0.0)	0 (0.0)	0 (0.0)		
Occupation								24.895	0.041
Farmer	63 (45.3)	32 (23.0)	14 (10.1)	10 (7.2)	5 (3.6)	1 (0.7)	1 (0.7)		
Worker/clerk	47 (33.8)	15 (10.8)	27 (19.4)	4 (2.9)	0 (0.0)	1 (0.7)	0 (0.0)		
Student	14 (10.1)	7 (5.0)	5 (3.6)	1 (0.7)	0 (0.0)	0 (0.0)	1 (0.7)		
Commercial services	15 (10.8)	4 (2.9)	10 (7.2)	1 (0.7)	1 (0.7)	0 (0.0)	0 (0.0)		
Marital status								26.271	0.001
Married	81 (58.3)	40 (28.8)	22 (15.8)	12 (8.6)	4 (2.9)	2 (1.4)	1 (0.7)		
Unmarried	47 (33.8)	11 (7.9)	31 (22.3)	4 (2.9)	0 (0.0)	0 (0.0)	1 (0.7)		
Divorced/widowed	11 (7.9)	6 (4.3)	3 (2.2)	0 (0.0)	2 (1.4)	0 (0.0)	0 (0.0)		
Ethnicity								24.641	<0.001
Han	125 (89.9)	55 (39.6)	53 (38.1)	8 (5.8)	6 (4.3)	2 (1.4)	1 (0.7)		
Minority	14 (10.1)	2 (1.4)	3 (2.2)	8 (5.8)	0 (0.0)	0 (0.0)	1 (0.7)		
Educational level								43.445	<0.001
College and above	29 (20.9)	6 (4.3)	22 (15.8)	0 (0.0)	0 (0.0)	1 (0.7)	0 (0.0)		
Middle school	85 (61.1)	38 (27.3)	32 (23.0)	7 (5.0)	6 (4.3)	1 (0.7)	1 (0.7)		
Primary school and below	25 (18.0)	13 (9.4)	2 (1.4)	9 (6.5)	0 (0.0)	0 (0.0)	1 (0.7)		

*URFs* unique recombinant forms

^a^Non parametric Kruskal–Wallis test was applied

^b^Compared HIV-1 subtype distribution among sexual contact, MTCT, IDU and blood

^c^Compared HIV-1 subtype distribution between heterosexual and homosexual

### HIV-1 genotype analysis

**Fig. 2:**
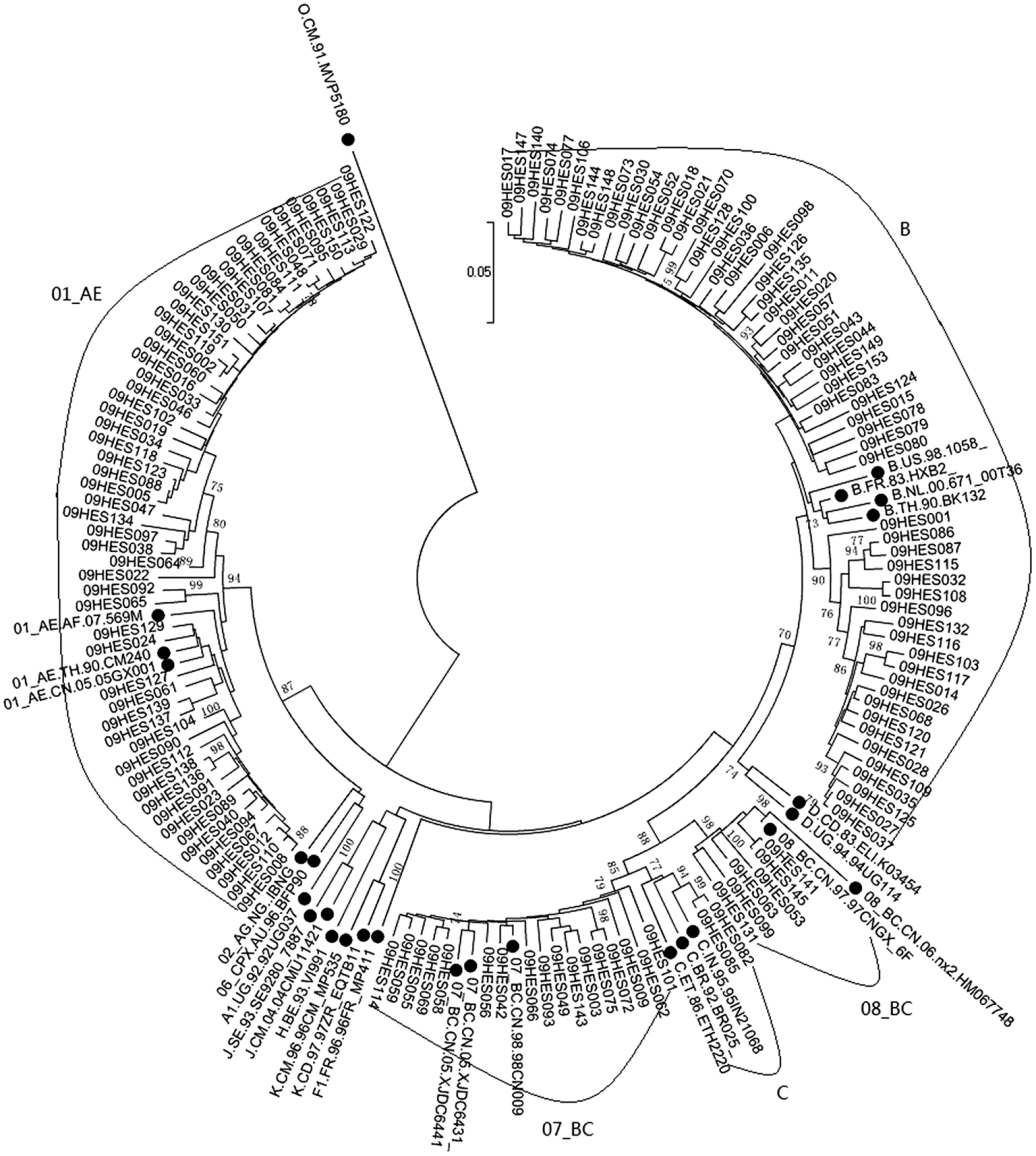
Neighbor-joining phylogenetic tree of partial *gag* gene sequences from newly diagnosed HIV-1 individuals. *Black dots* represent reference sequences; *Black square blocks* represent *gag* gene sequences of URFs

**Fig. 3:**
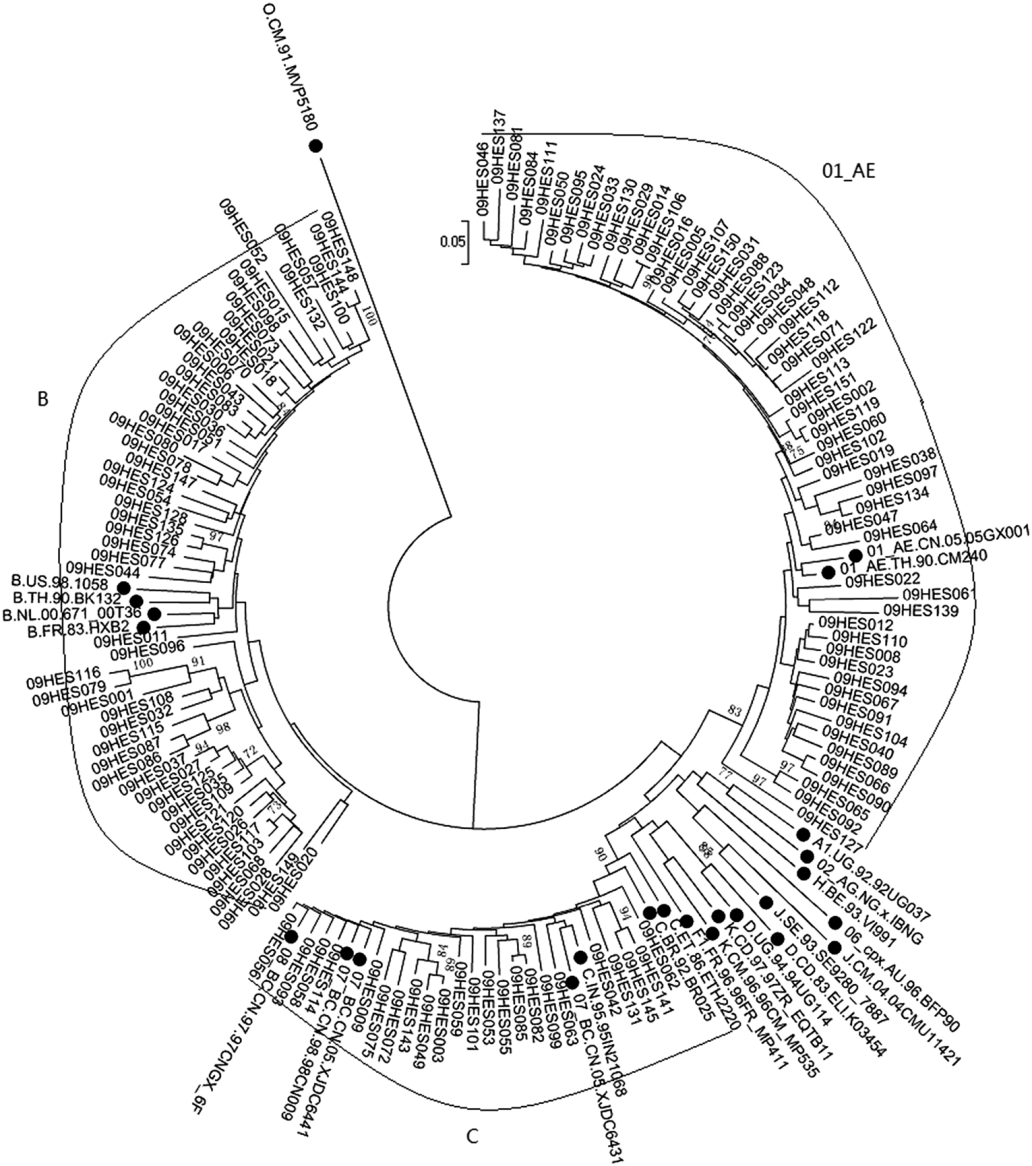
Neighbor-joining phylogenetic tree of partial *env* gene sequences from newly diagnosed HIV-1 individuals. *Black dots* represent reference sequences; *Black square blocks* represent *env* gene sequences of URFs

**Fig. 4:**
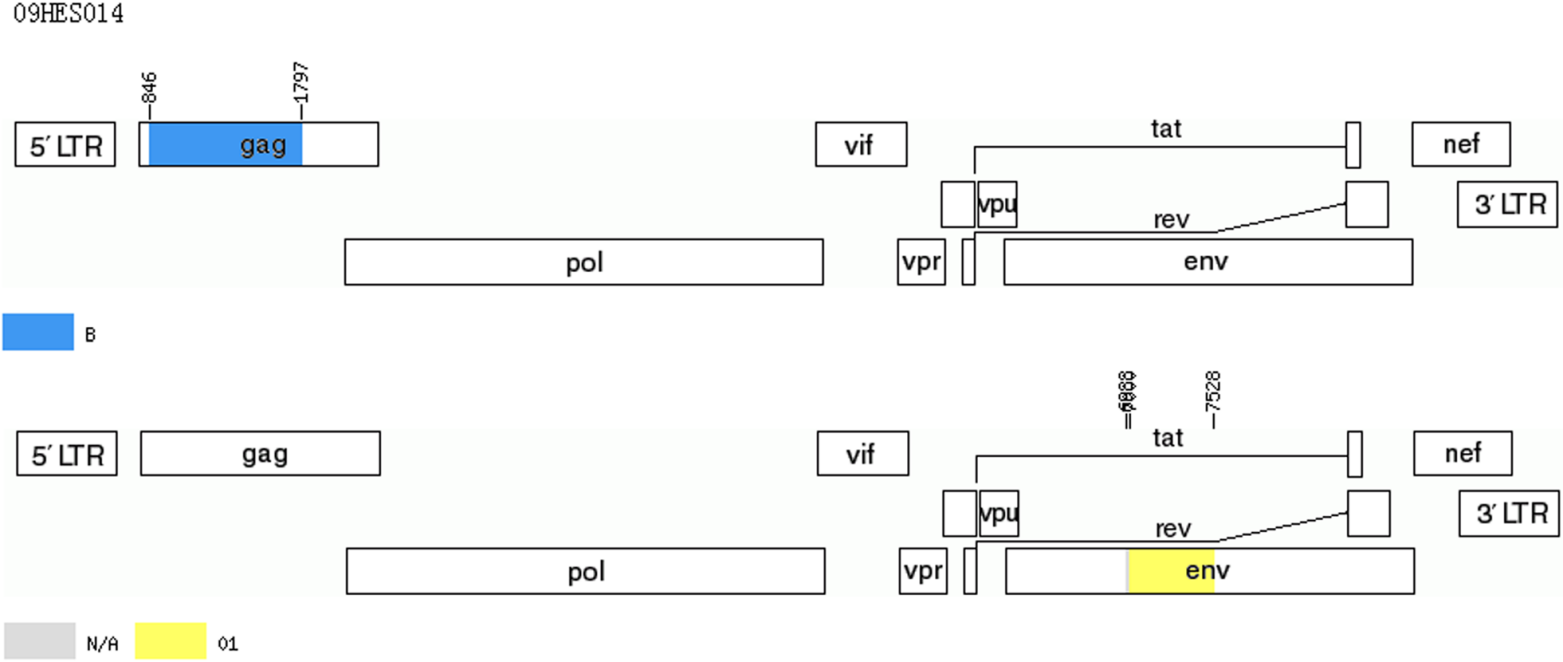
Genomic map of unique recombinant forms (URFs) (09HES014). The mosaic maps were generated using the jpHMM program (http://jphmm.gobics.de/submission_hiv.html)

**Fig. 5:**
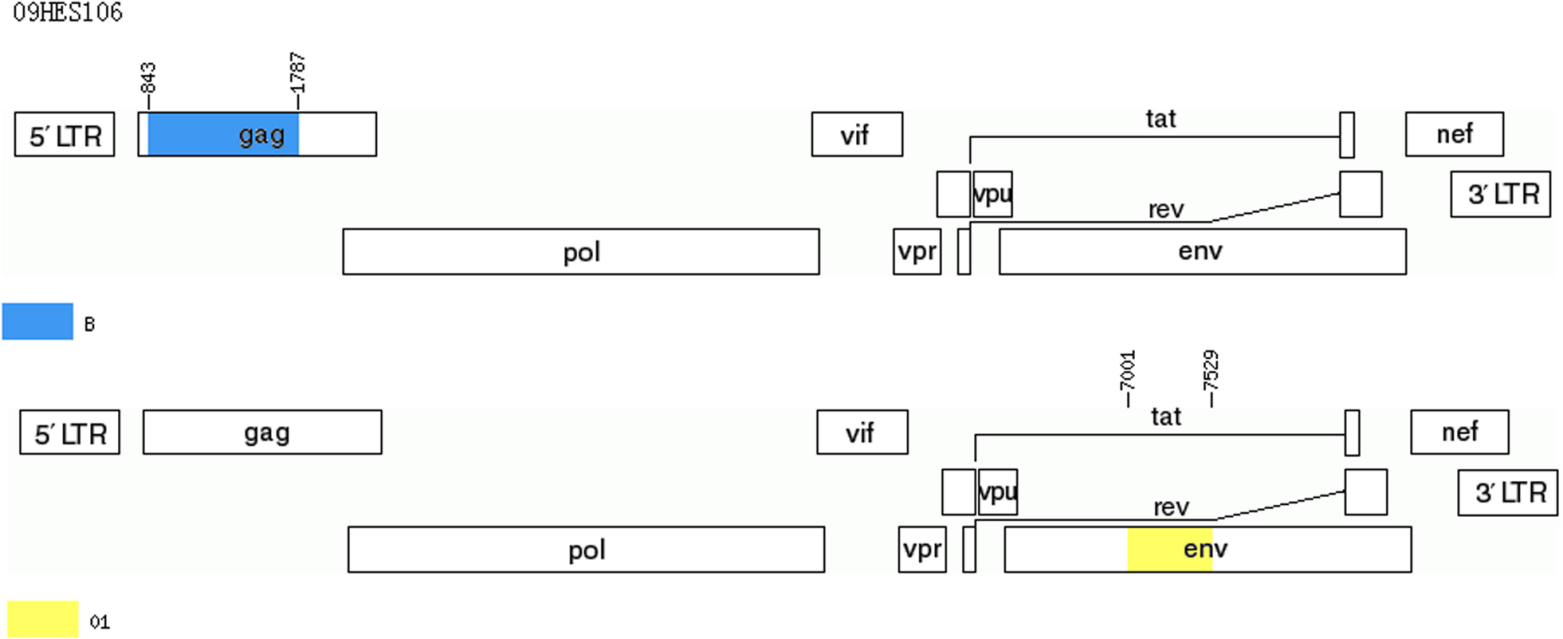
Genomic map of unique recombinant forms (URFs) (09HES106). The mosaic maps were generated using the jpHMM program (http://jphmm.gobics.de/submission_hiv.html)

Combined analyses of phylogenetic trees of *gag* and *env* gene sequences (Figs. 2 and 3) revealed six HIV-1 subtypes, including subtype B, subtype C, CRF07_BC, CRF08_BC, CRF01_AE and URFs. Subtype B (41.0 %, 57/139) was the most frequent subtype, followed by CRF01_AE (40.3 %, 56/139), CRF07_BC (11.5 %, 16/139), CRF08_BC (4.3 %, 6/139), URFs (1.4 %, 2/139) and subtype C (1.4 %, 2/139). The two URFs had the same recombination pattern: CRF01_AE/B (Figs. 4 and 5).

As shown in Table 3, for the *gag* and *env* gene regions, the mean genetic distances within the subtype B group were significantly greater than those within the CRF01_AE or CRF07_BC groups (*P* < 0.05). The genetic distances within the CRF01_AE group were almost equal to those within the CRF07_BC group. These phenomena suggested that the duration of the HIV-1 subtype B epidemic in Hebei was longer than those of CRF07_BC or CRF01_AE, and the initial circulating time of CRF07_BC was similar to that of CRF01_AE.

**Table 3:** Genetic distances among sequences belonging to different genotypes

Gene	Genetic distances (mean ± SE)		
	Subtype B (cases)	CRF01_AE (cases)	CRF07_BC (cases)
*gag*	0.055 ± 0.005 (n = 58)	0.043 ± 0.004 (n = 57)	0.039 ± 0.003 (n = 17)
*env*	0.190 ± 0.011 (n = 56)	0.115 ± 0.008 (n = 56)	0.116 ± 0.009 (n = 22)

### Demographic distribution characteristics of HIV-1 genotypes

To better describe the distribution characteristics of HIV-1 genotypes, a detailed demographic investigation was performed. As revealed in Table 2, the HIV-1 genotype distribution showed significant statistical differences in different demographic characteristics (gender, age, CD4 counts, source, transmission routes, occupation, marital status, ethnicity and educational level), especially in source, transmission routes, occupation and ethnicity. Both subtype B and CRF01_AE were distributed throughout almost all demographic characteristics. Of the nine infection sources, MSM was the major infection source, accounting for 20.9 % (29/139), followed by inpatient (18.0 %, 25/139), close contact (16.5 %, 23/139) and blood donor (12.9 %, 18/139). Five of the six subtypes were found in inpatients and close contacts, except for URFs in inpatient and subtype C in close contacts. With the exception of URFs and subtype C in preoperative detecting and CRF08_BC and subtype C in blood donors, four subtypes were detected in these two sources. Among the remaining sources, the number of HIV-1 subtypes was ≤3 in each source.

**Fig. 6:**
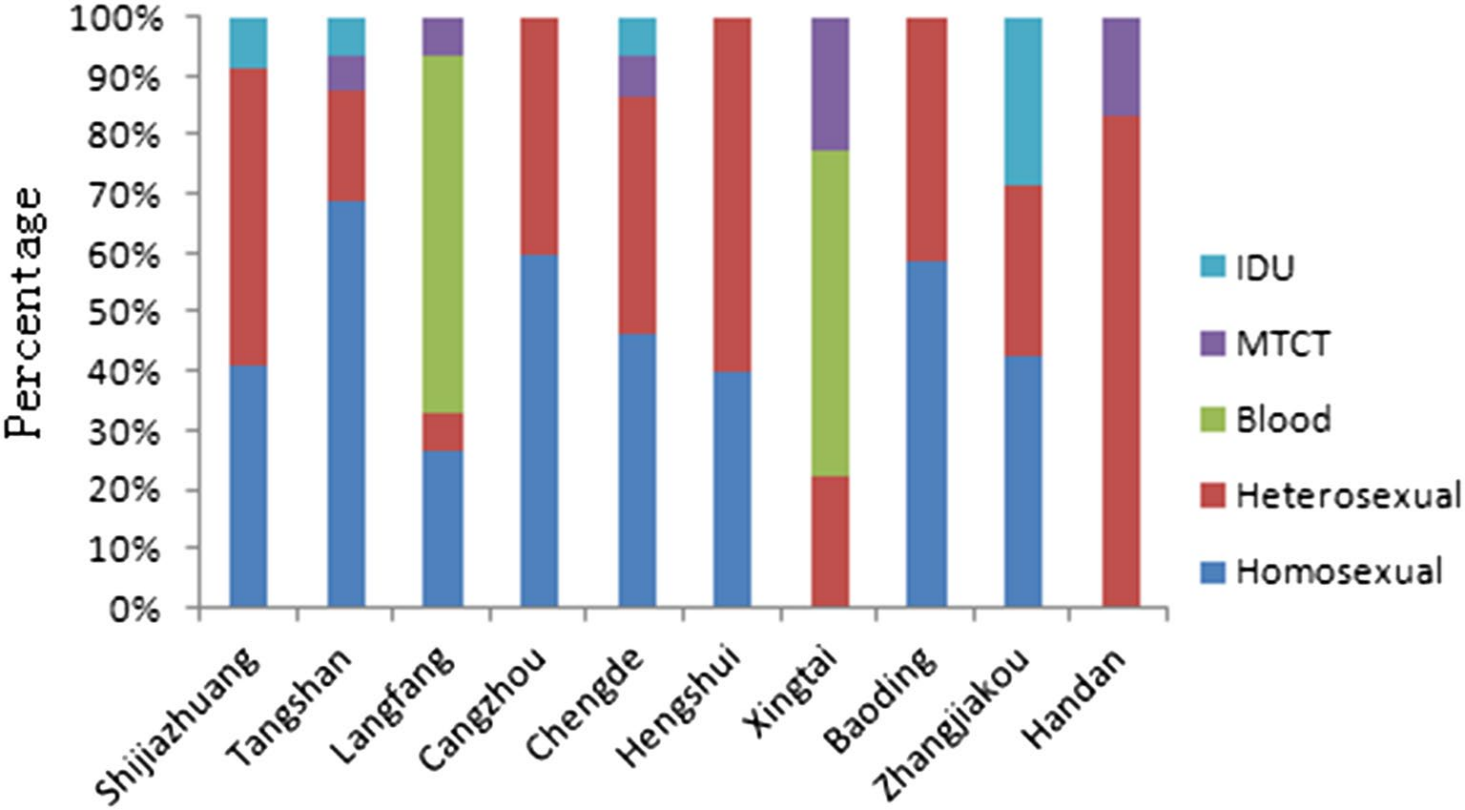
Distribution of HIV-1 transmission routes in ten prefectures of Hebei province

The occurrence of HIV-1 subtypes varied with different transmission routes, and significant statistical differences in HIV-1 genotype distribution were identified for different routes. For sexual contact, CRF01_AE was the most prominent genotype, accounting for 38.8 % (54/139), followed by subtype B (29.5 %, 41/139) and CRF07_BC (7.2 %, 10/139). All six subtypes were found in the heterosexual transmission subjects, but only three subtypes were found in the homosexual transmission subjects, and the HIV-1 genotype distribution showed a statistically significant difference between heterosexuals and homosexuals. In the blood transmission subjects, only subtype B was found. In IDUs, CRF07_BC accounted for 3.6 % (5/139), CRF08_BC for 1.4 % (2/139) and CRF01_AE for 0.7 % (1/139). In MTCT, subtype B accounted for 2.2 % (3/139), CRF07_BC for 0.7 % (1/139), CRF01_AE for 0.7 % (1/139) and subtype C for 0.7 % (1/139). Among the ten prefectures of Hebei, this statistical difference in HIV-1 genotype distribution for different routes was also revealed. As shown in Fig. 6 and Table 4, blood transmission accounted for 64.3 % (9/14) in Langfang and for 62.5 % (5/8) in Xingtai. Correspondingly, the proportions of subtype B in the subjects obtained from Langfang and Xingtai were 78.6 % (11/14) and 75.0 % (6/8), respectively. Sexual contact accounted for 91.1 % (41/45) in Shijiazhuang, 100.0 % (10/10) in Cangzhou, 100.0 % (13/13) in Chengde, 83.3 % (5/6) in Handan, 93.3 % (14/15) in Tangshan, 100.0 % (4/4) in Hengshui, 100.0 % (17/17) in Baoding and 71.4 % (5/7) in Zhangjiakou. Obviously, the proportion of sexual contact was significantly higher than other transmission routes in these eight prefectures. Correspondingly, the CRF01_AE strain and the subtype B strain, which were prevalent in sexual contact (Table 2), became the dominant strains in these eight prefectures (Table 4).

**Table 4:** HIV-1 genotype distribution of study subjects in ten prefectures

City	Subjects (%)	B (%)	CRF01_AE (%)	CRF07_BC (%)	CRF08_BC (%)	URFs (%)	C (%)	χ^2^	*P*
Baoding	17 (100)	10 (58.8)	7 (41.2)	0 (0.0)	0 (0.0)	0 (0.0)	0 (0.0)	4.106	0.483
Cangzhou	10 (100)	2 (20.0)	5 (50.0)	2 (20.0)	0 (0.0)	1 (10.0)	0 (0.0)	7.053	0.179
Chengde	13 (100)	6 (46.2)	5 (38.5)	1 (7.7)	1 (7.7)	0 (0.0)	0 (0.0)	1.845	0.872
Handan	6 (100)	2 (33.3)	1 (16.7)	3 (50.0)	0 (0.0)	0 (0.0)	0 (0.0)	8.040	0.157
Hengshui	4 (100)	1 (25.0)	3 (75)	0 (0.0)	0 (0.0)	0 (0.0)	0 (0.0)	4.100	0.725
Langfang	14 (100)	11 (78.6)	3 (21.4)	0 (0.0)	0 (0.0)	0 (0.0)	0 (0.0)	7.821	0.122
Shijiazhuang	45 (100)	15 (33.3)	17 (37.8)	7 (15.6)	5 (11.1)	1 (2.2)	0 (0.0)	9.567	0.046
Tangshan	15 (100)	3 (20.0)	12 (80.0)	0 (0.0)	0 (0.0)	0 (0.0)	0 (0.0)	9.434	0.067
Xingtai	8 (100)	6 (75.0)	0 (0.0)	0 (0.0)	0 (0.0)	0 (0.0)	2 (25.0)	18.816	0.001
Zhangjiakou	7 (100)	1 (14.3)	3 (42.9)	3 (42.9)	0 (0.0)	0 (0.0)	0 (0.0)	7.451	0.174

The distribution of HIV-1 genotypes in Han ethnicity and minority nationalities showed a significant statistical difference. All six subtypes were found in the studied Han ethnicity and four subtypes, except CRF08_BC and URFs, were found in the minority nationalities, mainly because subtypes CRF08_BC and URFs were confined to Han ethnicity in this study (Table 2). Additionally, 50.0 % (7/14) of the minority nationalities were the Yi who came from Liangshan and Sichuang. These subjects were illiterate female marriage immigrants who had been infected via the heterosexual or IDU routes with CRF07_BC (six cases) and subtype C (one case), suggesting that imported cases of HIV/AIDS have become one of the factors of HIV spread in Hebei (Table 5).

**Table 5:** Demographic characteristic of the ethinic minority population in this study

Sample code	Gender	Age	Infection source	Current address	Transmission routes	*Gag*	*Env*	Genotype	Occupation	Marital status	Ethnicity	Educational level	CD4 count
09HES055	Male	23	Inpatient	Shijiazhuang	Heterosexual	07-BC	C	07-BC	Farmer	Married	Dai	primary school	515
09HES056	Female	26	Female marriage immigrant^a^	Chengde	Heterosexual +IDU	07-BC	C	07-BC	Farmer	Married	Yi	Illiterate	428
09HES058	Female	38	Female marriage immigrant	Shijiazhuang	Heterosexual +IDU	07-BC	C	07-BC	Farmer	Married	Yi	Illiterate	406
09HES059	Female	32	Female marriage immigrant	Shijiazhuang	Heterosexual	07-BC	C	07-BC	Farmer	Married	Yi	Illiterate	248
09HES069	Female	25	Female marriage immigrant	Shijiazhuang	Heterosexual	07-BC	C	07-BC	Farmer	Married	Yi	Illiterate	546
09HES072	Female	26	Female marriage immigrant	Zhangjiakou	Heterosexual +IDU	07-BC	C	07-BC	Farmer	Married	Yi	Illiterate	275
09HES082	Female	30	Female marriage immigrant	Xingtai	Heterosexual	C	C	C	Farmer	Married	Yi	Illiterate	566
09HES093	Female	30	Female marriage immigrant	Cangzhou	Heterosexual	07-BC	C	07-BC	Farmer	Married	Yi	Illiterate	333
09HES101	Male	31	Spouse^b^	Cangzhou	Heterosexual	07-BC	C	07-BC	Farmer	Married	Uyghur	Illiterate	459
09HES067	Male	28	MSM	Shijiazhuang	Homosexual	01-AE	01-AE	01-AE	Teacher	Married	Oroqen	College	579
09HES078	Male	10	Close contact	Chengde	MTCT	B	B	B	Student	Unmarried	Man	Primary school	356
09HES081	Female	58	Spouse	Tangshan	Heterosexual	01-AE	01-AE	01-AE	Worker	Married	Man	Middle school	267
09HES094	Male	58	Inpatient	Tangshan	Homosexual	01-AE	01-AE	01-AE	Worker	Married	Hui	Middle school	255
09HES096	Male	33	MSM	Tangshan	Homosexual	B	B	B	Worker	Unmarried	Man	Middle school	297

^a^Young married women without local registered permanent residence

^b^The sexual partner of a person infected with HIV-1

Furthermore, our study showed that the patterns of HIV-1 genotype distribution were associated with the subjects’ occupations. 100 % (6/6) of HIV-1 subtypes were found in the farmers, but only 66.7 % (4/6) in workers, 66.7 % (4/6) in students and 66.7 % (4/6) in commercial services. This difference was mainly reflected in the differences in the prevalences of CRF08_BC, URFs and subtype C.

### Geographical distribution characteristic of HIV-1 subtypes

As shown in Table 4, the distribution of all six subtypes showed significant differences in Shijiazhuang and Xingtai, and no statistical difference in other prefectures (Baoding, Zhangjiakou, Handan, Hengshui, Cangzhou, Tangshan, Langfang and Chengde). The distribution of subtype B (χ^2^ = 21.753, *P* = 0.006), CRF01_AE (χ^2^ = 21.134, *P* = 0.008), CRF07_BC (χ^2^ = 18.552, *P* = 0.006) and subtype C (χ^2^ = 14.311, *P* = 0.013) showed significant differences in all 10 prefectures. However, the distribution of URFs (χ^2^ = 8.757, *P* = 0.620) and CRF08_BC (χ^2^ = 5.692, *P* = 0.728) showed no statistical significance, suggesting that these two subtypes are randomly distributed in the ten prefectures.

Subtype B was distributed throughout the ten prefectures, and CRF01_AE was distributed in nine prefectures, except Xingtai. CRF07_BC and CRF08_BC, which previously had been mainly prevalent in IDUs, were found in heterosexuals, MTCT or IDUs in this study. CRF07_BC and CRF08_BC was distributed in both IDUs and heterosexuals in Shijiazhuang, IDUs in Zhangjiakou, both IDUs and MTCTs in Chengde, and heterosexuals in Cangzhou. Two URFs were found in subjects infected by heterosexual contact in Shijiazhuang and Cangzhou. Two subjects infected with subtype C came from Xingtai and were infected through heterosexual contact (Yi nationality, female marriage immigrant) and MTCT, respectively. Five of six subtypes, except subtype C, were found in Shijiazhuang. Four of six subtypes, except subtype C and CRF08_BC, were found in Cangzhou, and in four of six subtypes, except URFs and subtype C, in Chengde. In other prefectures, three or fewer subtypes were found.

## Discussion

In this study, to evaluate HIV-1 genetic diversity and its distribution characteristics in Hebei, the first molecular epidemiological investigation of HIV-1 was conducted among newly diagnosed individuals. The overall analyses of HIV-1 *gag* and *env* gene sequences revealed two subtypes, three CRFs and one URF. We found that subtype B was the most frequent subtype. Historically, subtype B was responsible for the first epidemic among paid blood donors in Langfang and Xingtai, where an HIV-1 outbreak occurred through blood transmission between 1993 and 1995. A previous report indicated that subtype B was confined in these prefectures [7, 8, 13]. In this study, subtype B, including next generation cases [8] (via transmission between spouses or MTCT), still predominated in Langfang and Xingtai. Some reports [14–16] indicated that the HIV-1 mutation rate was 0–2 % when HIV-1 began to circulate in an area, and HIV-1 mutated at a rate of 0.5–1 % per year. The subjects in this study were collected in 2011 and the initial circulating time of subtype B was between 1993 and 1995. Therefore, we inferred from *env* gene distances (19.0 %) that subtype B has been circulating for 17–19 years, which was consistent with the known epidemiology [8]. CRF01_AE was the second most frequent genotype. According to previous reports, CRF01_AE strains in China were found in intravenous drug users (IDUs) for the first time in Yunnan, then spreading to Liaoning along the southeast coast of China via drug injection and sexual contact [17, 18], and spreading finally to Hebei from Liaoning [19]. The first CRF07_BC epidemic and the first subtype C epidemic were initiated by one IDU from Guangxi and two overseas workers returning from abroad in 2002 [10], respectively. To date, the prevalence of CRF07_BC has risen to 11.5 %. The influx into Hebei of 42.9 % (6/14) of CRF07_BC occurred through female marriage migrants (Yi) infected by heterosexual/IDUs, who were of Yi ethnicity, and came from Liangshan and Sichuan. It is believed that the Yi engage in casual sex, have concurrent sexual partnerships and inject drugs [20, 21], which have resulted in a higher HIV/AIDS prevalence (4.63 %) among the Yi [22] than the overall prevalence (0.058 %) in China [23]. This phenomenon suggests that the imported cases of HIV/AIDS represent one of the causes of HIV genetic diversity in Hebei.

Since the first case was found in 1989, more HIV-1 subtypes have been identified in Hebei, from three subtypes identified in 2002 to the six subtypes identified in this study, which is in accord with the major HIV-1 genotypes of China [24]. However, the prevalence of different genotypes has changed continually from 2002 to 2011 (this study), especially for CRF01_AE (from 29.4 % in 2007 to 40.3 % in 2011) and subtype B (from 61.8 % in 2002 to 41.0 % in 2011). These changes were closely associated with the transition of transmission routes. In Hebei, HIV-1 individuals infected by blood accounted for 84.4 % between 1989 and 2003. However, from 2004 to 2009, the percentage of HIV-1 individuals infected by blood decreased sharply from 61.9 % to 9.9 %. Conversely, that of HIV-1 individuals infected by sexual contact increased sharply from 23.8 % to 82.5 %, especially homosexual transmission, which increased from 0.68 % to 40.9 % [9], consistent with the changing trend of HIV-1 subtypes in China [25]. Our study confirmed these significant differences in HIV-1 genotype distribution among different transmission routes by analyzing the geographical distribution characteristics of HIV-1 subtypes. Additionally, the HIV-1 genotype distribution showed significant differences in all demographic characteristics, such as occupation, source and ethnicity. We inferred that the changing trend of transmission routes among these demographic characteristics also played a critical role. Therefore, compared with the lack of statistically significant differences of HIV-1 subtypes among different transmission routes in Yunnan [26], in Hebei, the specificity and changing trend of HIV-1 transmission routes resulted in differences in HIV-1 genotype distribution in different areas or different demographic characteristics in Hebei. In particular, all six subtypes were found in the heterosexual transmission cases, showing that HIV-1 is spreading from high-risk populations to the general populations in Hebei, China [6]. In addition, CRF01_AE, instead of subtype B, has become the major strain of HIV-1 infection among homosexuals.

In this work, although only HIV-1 subtype B was spread through blood transfusion (Table 2), subtype B was found not only in blood, but also MTCT and sexual contact cases and was distributed throughout the ten prefectures. We hypothesized two possible explanations: one was next generation transmission among HIV-1 individuals with subtype B (via family transmission and disease transmission between couples, as well as between mothers and infants). Indeed, Chen et al. reported that among blood donors, there was a couples transmission rate of 11.3 % and an MTCT rate of 38.5 % within 18 years after HIV-1 subtype B infection [8]. The other explanation was that subtype B has spread from the high-risk population to the general population by different high-risk behaviors, especially sexual contact, associated with the growing size of the floating population.

Moreover, CRF01_AE and subtype B constituted the two most frequent genotypes in Hebei, which is distinct from some provinces in northwestern and southwestern China, where CRF08_BC and CRF07_BC ware the most common genotypes [6]. As a result of co-circulation and dual-infection involving subtype B and CRF01_AE, we found two CRF01_AE/B (URFs) strains in this study. According to our previous reports [10, 11, 13, 19], URFs were first identified in Hebei. The prevalence (1.4 %) of URFs was lower than that in China’s other provinces [27, 28]. Two specimens with URFs (one an inpatient, the other a blood donor) were collected from Shijiazhuang and Cangzhou, respectively, where the sexual transmission rate was more than 90 %. Therefore, the occurrence of URFs was attributed to the complexity of high-risk behaviors and HIV-1 genetic diversity in Hebei, which suggests that research into URFs should be a priority for future studies, providing the critical data for early prevention and control of HIV-1.

## Conclusions

Six HIV-1 subtypes were identified among newly diagnosed HIV-1 individuals in Hebei Province, and the distribution of these subtypes showed significant demographic and geographical differences. These differences were closely associated with transmission routes. All six subtypes were found in sexual contact cases, showing that HIV-1 is spreading from the high-risk populations to the general populations in Hebei. Currently, as with the HIV-1 epidemic situation in the rest of China, sexual contact transmission has been the most frequent transmission route of HIV in Hebei. Consequently, our study revealed the evolution and genotype distribution of HIV-1 by investigating newly diagnosed HIV-1 individuals in Hebei, China, which provides important information for formulating a strategic plan for HIV prevention and control in China.

## Abbreviations

HIV-1: human immunodeficiency virus type one
MSM: men who have sex with men
VCT: Voluntary Counseling and Testing
MTCT: mother-to-child
IDU: intravenous drug injection
URFs: unique recombinant forms
CRF: circulating recombinant form
